# Impact of age on the seasonal prevalence of hypernatremia in the emergency department: a single-center study

**DOI:** 10.1186/s12245-019-0246-7

**Published:** 2019-09-18

**Authors:** Naohiko Imai, Hirofumi Sumi, Yugo Shibagaki

**Affiliations:** Division of Nephrology and Hypertension, Department of Internal Medicine, St. Marianna University School of Medicine, 2-16-1 Sugao, Miyamae-ku, Kawasaki, Kanagawa Japan

**Keywords:** Hypernatremia, Seasonal, Prevalence, Emergency department

## Abstract

**Background:**

Hypernatremia is one of the most commonly encountered electrolyte disorders in the emergency department (ED). Few studies have reported the seasonal fluctuations of the prevalence of hypernatremia with conflicting results. We investigated the seasonal prevalence of hypernatremia in an emergency department in Japan.

**Methods:**

A total of 12,598 patients presented to the ED between January 2015 and December 2017 were reviewed. The adult group aged between 18 and 64 years old consisted of 5427 patients and the elderly group aged over 65 years consisted of 7171 patients. Information collected included age, sex, serum sodium, and serum creatinine. Hypernatremia was defined as a serum sodium leve1 > 145 mEq/L, and moderate to severe hypernatremia was defined as a serum sodium level ≥ 150 mEq/L.

**Results:**

The prevalence of hypernatremia was significantly higher in the elderly group than in the adult group (2.6% vs. 0.7%; *p* < 0.001). Similarly, the prevalence of moderate to severe hypernatremia was significantly higher in the elderly group than in the adult group (1.0% vs. 0.1%; *p* < 0.001). The prevalence of hypernatremia and moderate to severe hypernatremia was significantly higher in the elderly group than in the adult group in all seasons. In the elderly group, the seasonal prevalence of moderate to severe hypernatremia was significantly higher during the winter. Also, there was a correlation between weather temperature and the prevalence of moderate to severe hypernatremia in the elderly group (*r* = − 0.34, *p* = 0.04).

**Conclusions:**

Hypernatremia is prevalent in the elderly and the prevalence is highest during the winter. Special attention should be paid in the elderly patients to prevent hypernatremia especially in the winter.

## Introduction

Hypernatremia is one of the most commonly encountered electrolyte disorders in the emergency department and is known for its high morbidity and mortality [1–6]. Age is a strong independent risk factor of hypernatremia [1–6]. Considering the aging population in Japan, the increased susceptibility of the elderly to develop hypernatremia is particularly important. Although several studies have reported the prevalence of hypernatremia in patients admitted to the emergency department, few studies have reported the seasonal fluctuations of the prevalence of hypernatremia [4, 7]. Furthermore, the impact of age on the seasonal fluctuations on the prevalence of hypernatremia has not been reported in Japan. Thus, we investigated the impact of age on the seasonal prevalence of hypernatremia in an emergency department in Japan.

## Materials and methods

All adult patients (18 years old or older) who had their serum sodium levels measured at the emergency department of between January 2015 and December 2017 were included. Hypernatremia was defined as a serum sodium leve1 > 145 mEq/L, and moderate to severe hypernatremia was defined as a serum sodium level ≥ 150 mEq/L. Information collected included age, sex, serum sodium, and serum creatinine. The eGFR of each participant was calculated using the following formula: eGFR (mL/min/1.73 m^2^) = 194 × serum creatinine^−1.094^ × Age^−0.287^ × 0.739 (if female) [8]. We used the meteorological parameters provided by the Japan Meteorological Agency. The seasons are defined as follows: spring: March–May, summer: June–August, fall: September–November, and winter: December–February. This single-center study was approved by the Institutional Review Boards at our institution.

### Statistical analyses

Data analysis was performed using SPSS, Version 21.0 (IBM Corp, Armonk, NY). The results were expressed as mean and standard deviation for continuous variable unless otherwise specified. Student *t* test or analyses of variance were used to compare means for continuous variables. Chi-square tests were used to test statistical differences for categorical variables. A *p* value of < 0.05 was considered statistically significant.

## Results

### Patients’ characteristics

From January 2015 to December 2017, a total of 12,598 adult patients had their serum sodium measured at the emergency department. The key characteristics of the patients are summarized (Table 1). The mean age of the patients was 63.6 ± 22.3 years and 49.5% of patients were male. Patients were divided into the adult group aged from 18 to 64 years and elderly group aged 65 years and over. The mean age of the adult group (*N* = 5427) was 41.3 ± 13.7 years and 50.9% of patients were male. The mean age of the elderly group (*N* = 7171) was 80.6 ± 8.3 years and 48.4% of patients were male.

**Table 1:**
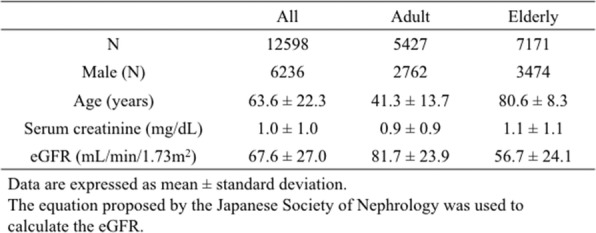
Patients characteristics

### Prevalence of hypernatremia and moderate to severe hypernatremia

The prevalence of hypernatremia was 1.8% and the prevalence of moderate to severe hypernatremia was 0.6% (Table 2). The prevalence of hypernatremia was significantly higher in the elderly group than in the adult group (2.6% vs. 0.7%; *p* < 0.001). Similarly, the prevalence of moderate to severe hypernatremia was significantly higher in the elderly group than in the adult group (1.0% vs. 0.1%; *p* < 0.001).

**Table 2:**
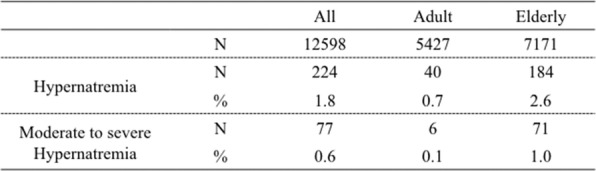
Prevalence of hypernatremia and moderate to severe hypernatremia

### Seasonal prevalence of hypernatremia and severe hypernatremia

**Fig. 1:**
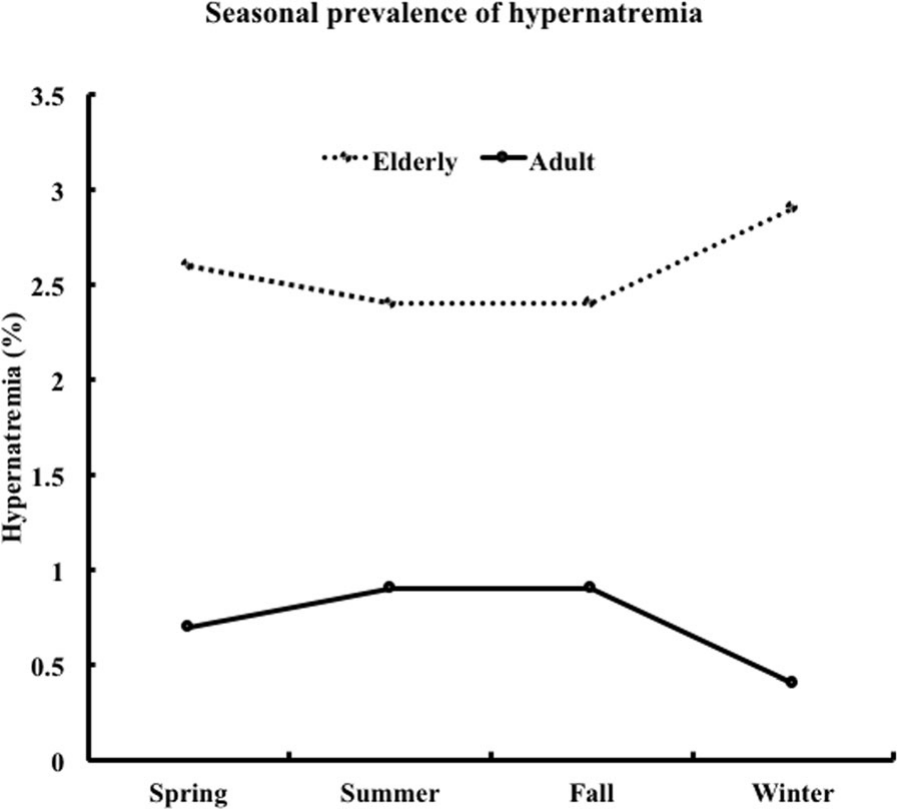
Seasonal prevalence of hypernatremia

**Fig. 2:**
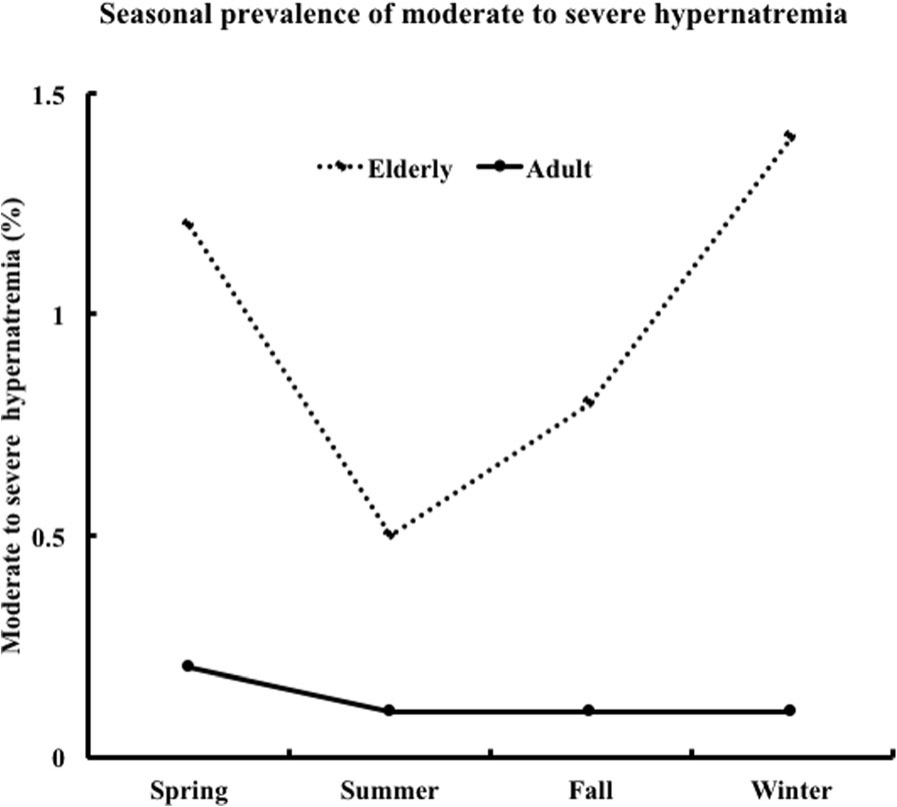
Seasonal prevalence of moderate to severe hypernatremia

The prevalence of hypernatremia was significantly higher in the elderly group than in the adult group in all seasons (Fig. 1). Similarly, the prevalence of moderate to severe hypernatremia was significantly higher in the elderly group than in the adult group in all seasons (Fig. 2). In the adult group, the prevalence of hypernatremia and moderate to severe hypernatremia did not differ between the seasons. In the elderly group, the prevalence of moderate to severe hypernatremia was significantly higher in winter (*p* = 0.04 vs. summer) (Table 3).

**Table 3:**
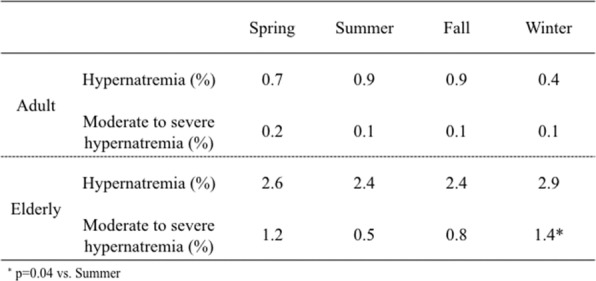
Seasonal prevalence of hypernatremia and moderate to severe hypernatrie in the adult and the elderly

### Monthly weather temperature and prevalence of moderate to severe hypernatremia

**Fig. 3:**
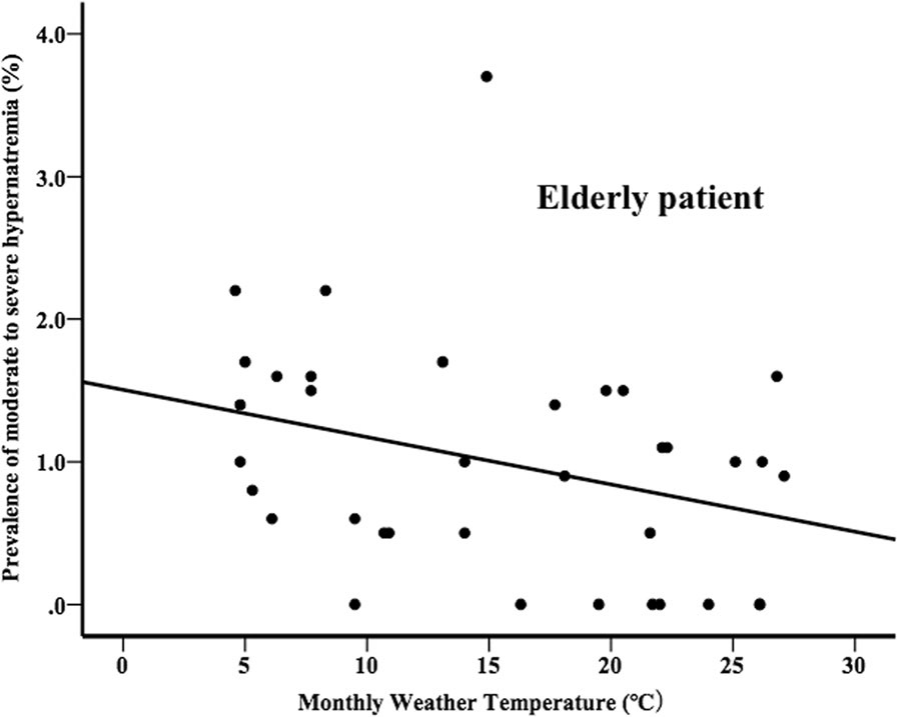
Relationship between prevalence of moderate to severe hypernatremia and monthly weather temperature in the elderly group

In the adult group, the monthly weather temperature did not show any correlation with monthly prevalence of moderate to severe hypernatremia (*r* = 0.236, *p* = 0.17). On the other hand, in the elderly group, the monthly weather temperature showed a linear correlation with monthly prevalence of moderate to severe hypernatremia (*r* = − 0.342, *p* = 0.04) (Fig. 3).

## Discussion

The prevalence of hypernatremia including moderate to severe hypernatremia was significantly higher in the elderly group compared to the adult group during the 3-year period. Also, the seasonal prevalence of hypernatremia including moderate to severe hypernatremia was significantly higher in the elderly group compared to the adult group during the 3-year period.

In our study, the prevalence of hypernatremia was 1.8% and the prevalence of moderate to severe hypernatremia was 0.6%, which was similar to the previous reported literature [1, 3, 4, 8] (Table 4). The prevalence varies depending on the population at risk and in our study, and the prevalence of hypernatremia in the adult group and the elderly group was 0.7% and 2.6%, respectively.

**Table 4:**
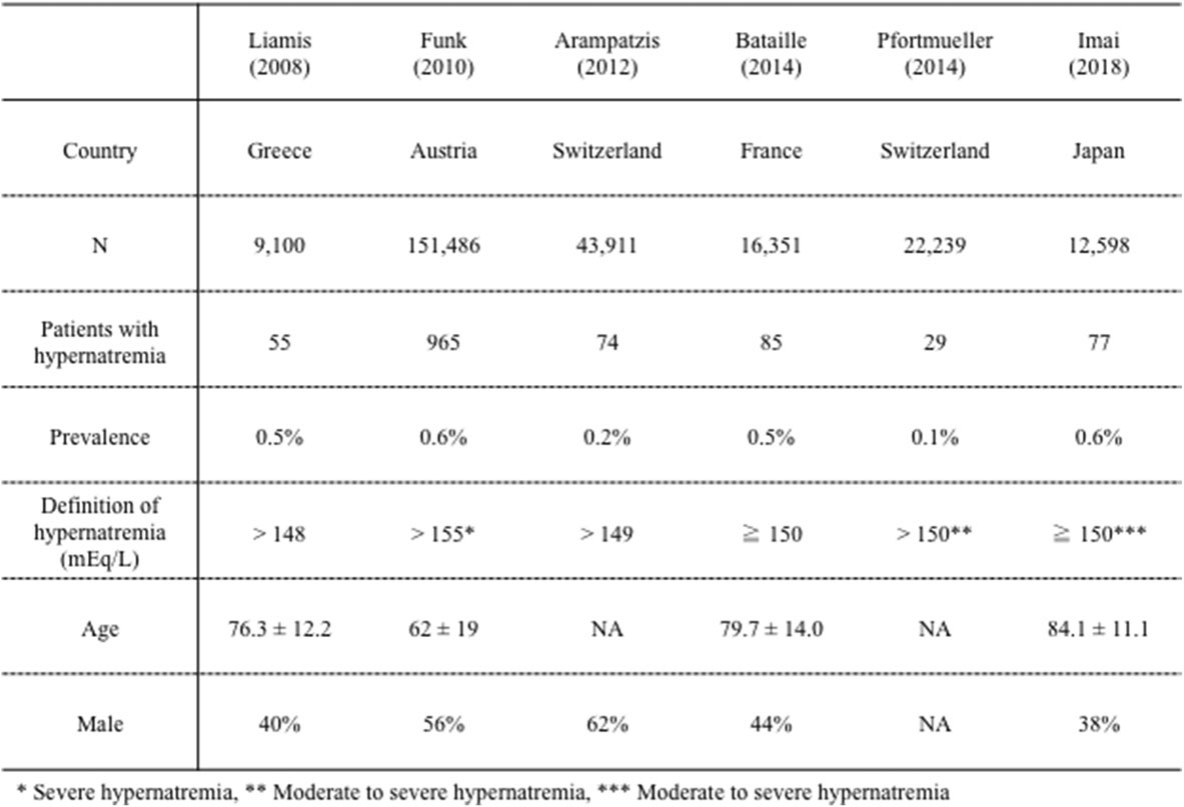
Prevalence and characteristics of out-of-hospital acquired hypernatremia

The prevalence of hypernatremia is strongly affected by age and is higher in elderly patients. It is important that physicians taking care of elderly patients be fully aware of the fact that elderly patients are susceptible to develop hypernatremia because the increased morbidity and mortality associated with hypernatremia cannot be ignored.

The prevalence of hypernatremia is affected by the weather temperature. Although hot weather is an understandable risk factor for hypernatremia, an increased prevalence of hypernatremia has been reported during the winter [4]. Our study confirmed this finding. In our study, the prevalence of hypernatremia was highest during the winter only in the elderly group. This is contrary to hyponatremia whose prevalence is highest during the summer [9, 10].

Although water intake and loss are approximately 40% higher in the summer than in the winter, in the elderly group, the prevalence of moderate to severe hypernatremia was highest in the winter [11]. This might be because less important care is given to the patient’s hydration status in winter than in summer [12]. The correlation between the prevalence of hyponatremia and the monthly weather temperature has been reported, but the correlation between the prevalence of hypernatremia and the monthly weather temperature has not been reported [9, 10]. For the first time, our study showed a correlation between the prevalence of moderate to severe hypernatremia and the monthly weather temperature in the elderly patients. This correlation was not observed in the adult group. It should be emphasized that an adequate water intake is necessary for the elderly not only in the summer but also in the winter.

This study is not without limitations. First, this is a single-center study and there is a possibility of selection bias in the patients enrolled. Second, serum sodium was not corrected for plasma glucose levels, when elevated. Third, several potential confounders such as the reason for visit, medications which could induce hypernatremia, history of hypernatremia, other electrolyte disorders, and past medical history were not collected.

## Conclusion

We observed a major impact of age on the seasonal prevalence of hypernatremia. Elderly patients had a significantly higher seasonal prevalence of hypernatremia and moderate to severe hypernatremia compared to adult patients. In the elderly patients, the prevalence of moderate to severe hypernatremia was highest in the winter. Special attention should be paid in the elderly patients to prevent hypernatremia especially in the winter.

## Data Availability

The datasets used and/or analyzed during the current study available from the corresponding author on reasonable request.

## Abbreviation

ED: Emergency department
